# The Characteristics and HIV-Related Outcomes of People Living with Co-occurring HIV and Mental Health Conditions in the United States: A Systematic Review of Literature from 2016 to 2021

**DOI:** 10.1007/s10461-023-04150-9

**Published:** 2023-08-10

**Authors:** Thomas O’Grady, Nina Inman, Alitasha Younger, Bishan Huang, Taylor Olivia Bouton, Heeun Kim, Emily DeLorenzo

**Affiliations:** 1 New York State Department of Health, AIDS Institute, Albany, NY USA; 2 New York State Department of Health, Corning Tower, ESP, Room 760, Albany, NY 12237-0627 USA; 3 Department of Epidemiology and Biostatistics, University at Albany School of Public Health, 1 University Pl, Rensselaer, NY 12144 USA

**Keywords:** HIV/AIDS, Behavioral health, Syndemics, Health outcomes, Service utilization

## Abstract

Considering advances in HIV prevention and treatment, jurisdictional efforts to end the HIV/AIDS epidemic, and reduced stigma towards people living with HIV infection and mental health conditions, the authors systematically reviewed studies published between 2016 and 2021 and identified 45 studies that met the eligibility criteria. The review found that stigma towards mental health conditions still acts as a barrier to accessing HIV treatment, which impacts treatment outcomes. Additionally, social determinants of health, such as housing instability and poverty, appear to impact mental health and, therefore, HIV-related outcomes. The review also highlighted the mutually reinforcing effects of HIV, mental health, and substance use conditions, providing valuable insights into the syndemic effects of these co-occurring conditions. Overall, the review highlights the need to address stigma and social determinants of health in HIV prevention and treatment efforts and to integrate mental health services into HIV care to improve outcomes for people living with both HIV and mental health conditions.

## Introduction

The HIV epidemic remains a prominent public health issue within the United States. In 2020, 30,635 people in the United States were diagnosed with HIV, with the impact of HIV disproportionately affecting MSM and Black/African American communities [1]. Although there is no cure for HIV, treatment is available to achieve VLS, resulting in increased QoL and the prevention of HIV transmission to others [2].

PLWH have disproportionately experienced syndemic factors including mood disorders, anxiety, drug use, unhealthy alcohol use, and poorer QoL relative to the general population [3–5]. Mental health conditions are more common among PLWH due to the stress of living with a serious condition, potential loss of social support, and experiences of HIV/AIDS stigma and discrimination amongst the general population [6]. It is estimated that 63% of PLWH have a co-occurring mental health condition, in comparison to 31% of people not living with an HIV diagnosis [7]. Research demonstrates that people with HIV are more likely to experience multiple, co-occurring mental illnesses and substance use disorders—collectively referred to as behavioral health conditions [8]. Co-occurring HIV and behavioral health conditions are associated with lower engagement in HIV care and treatment and poorer health outcomes, including low viral load suppression and mortality [9].

Previous studies in developing countries, as well as in the United Kingdom, and the United States have examined the relationship between mental disorders, behavioral health conditions, and HIV outcomes. Researchers found that identifying as female gender, being poor and unemployed, poor overall health status, inadequate care, and lack of social support were among the factors that increased psychiatric comorbidities in PLWH [10]. PLWH with co-occurring mental illness had worse quality of life (QoL) due to lower ART utilization, decreased ART adherence, and immunologic change associated with the mental illness [11]. Meanwhile, substance use and aging that co-occurred with psychiatric symptoms may further decrease HIV treatment adherence and hinder the effectiveness of pharmacological or psychosocial interventions [12–14]. However, few studies have systematically reviewed the topic area in recent years in the U.S. context despite critical changes in HIV management and strategic planning especially following both state and federal ending the HIV Epidemic initiatives in the US [15, 16]. A better understanding of the recent characteristics and service utilization of people living with co-occurring HIV and mental and behavioral health conditions will allow for the creation of targeted interventions for this population, improving HIV and mental and behavioral health outcomes, QoL, and the elimination of health disparities and inequities. This paper presents a systematic review of the literature from 2016 to 2021 on co-occurring mental and behavioral health conditions among PLWH in the United States with the goal of learning more about how co-occurring mental and behavioral health conditions impact HIV-related health outcomes.

## Methods

The aim of this review is to describe the demographic and clinical characteristics, service utilization, and patterns of engagement in the HIV and behavioral healthcare continuum among people living with co-occurring HIV and mental health conditions in the United States. This review was developed in accordance with the PRISMA 2020 Statement and Explanation and Elaboration Document.

### Data Sources & Search Process

Original peer-reviewed articles published in English language journals from 2016 to October 2021 were obtained from systematic searches of the following databases: Access Medicine, EBSCO, OVID, PubMed, Scopus, Web of Science. The search was implemented in May 2022.

The search query consisted of terms such as (HIV, AIDS), (Mental Health, Mental Health Disorders, Mental Illness Anxiety, Behavioral Health, Depression, Psychiatric Inpatient, Schizophrenia, Serious Mental Illness, Suicide), (Retention in Care, Barriers to care, Access to care, Adherence, Viral Response, Viral Load, Viral Suppression, Antiretroviral Therapy), (Risk Factors, Co-occurring, Comorbid), (Quality of Life, Health Outcomes, Health Disparities, Quality of Care, Biobehavioral Transmission Risk, Syndemics, Hospitalization, Mortality, Death) and was tailored to the specific requirements of each database. Documents like reports, essays, commentaries, and grey literature were not considered as they did not undergo peer review.

All searches were conducted using “KW,” Author Identified Keywords. A secondary reference search was conducted on two systematic reviews identified in the search [17, 18]. Two results were yielded from this search strategy.

### Study Selection and Data Extraction

**Fig. 1:**
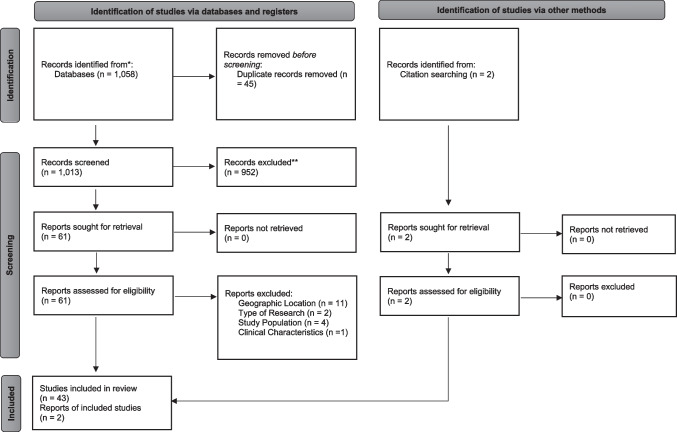
PRISMA 2020 flow diagram for new systematic reviews

All search results were compiled and screened by one reviewer (NI) for duplicates, removing 45 results. Next, two reviewers (HK, TB) independently applied inclusion and exclusion criteria to the title and abstract of each citation. To be included in this systematic review, original peer-reviewed articles had to meet the following criteria. The applied inclusion and exclusion criteria addressed six major areas: type of research, date of publication, geographic location, study population, clinical characteristics, and patient interventions (Table 1). Following the independent review of titles and abstracts, a third reviewer (NI) compared the inclusion/exclusion recommendations and resolved disagreements. This screening process resulted in the removal of 952 results. The inclusion and exclusion criteria were then applied to the text of the remaining 61 results (Fig. 1).

**Table 1:** Inclusion and exclusion criteria

	Inclusion criteria	Exclusion criteria
Type of research	Peer-reviewed literature	Abstracts/poster presentations, systematic reviews, meta-analysis, and policy reviews
Date of publication	January 1, 2016, to December 31, 2021	Studies published before January 1, 2016
Geographic location	United States	All other countries
Study population	Adults aged 18 and older living with co-occurring HIV and mental illness	Adolescents/children under 18 years, other populations
Clinical characteristics	HIV, anxiety disorders, bipolar and related disorders, depressive disorders, disruptive, impulse-control, and conduct disorders, dissociative disorders, feeding and eating disorders, gender dysphoria, obsessive–compulsive and related disorders, personality disorders, schizophrenia spectrum and other psychotic disorders, trauma- and stressor-related disorders	Cognitive impairments, suicidal ideation or attempts, other psycho-social factors
Patient interventions	None	Studies on the effects of specific medications

The reviewers used a two-step process to assess the articles based on eligibility criteria. In the first step, they selected articles based on title and abstract, and in the second step, they screened the full text of the included articles. If the title/abstract was insufficient, they retrieved and examined the full article before making a final decision. Conflicts and disagreements between the reviewers were discussed and resolved through consensus. Following the screening process and additions from the screened systematic reviews, 45 studies were included in the data extraction process. Reviewers (AY, ED, HK, NI, TB, TO) examined and summarized all remaining studies utilizing a standardized template (Table 2).

**Table 2:** Characteristics of studies

Authors	Year	Location	Goal	Sample size	Type of study	Key findings
Anderson, Monda, Haardörfer, and Waldrop-Valverde	2019	Atlanta, GA	To examine the prospective and reciprocal relationship between depressive symptoms and the patient–provider relationship	190	Prospective cohort	Findings suggest that there is no causal association between depressive symptoms and healthcare provider engagement. Results indicated that although the patient–provider relationship was beneficial for mental health outcomes in people living with HIV, addressing sociodemographic factors may be of greater importance
Angrand, Sperling, Roccobono, Osborne, and Jao	2018	NYC, NY	To compare the risks of antepartum depression between pregnant women with and without HIV as well as between pregnant women with HIV and HIV-uninfected women	245	Secondary analysis performed within a larger cohort study	Overall, women living with HIV had higher rates of depression than HIV-uninfected women, with pregnant women with HIV demonstrating a clinically and statistically significant increased risk compared to HIV-uninfected women. Observed rates of antepartum depression in HIV-infected pregnant women were lower than reported in previous studies, which ranged from 22 to 44%. In our control population of HIV-uninfected women, the observed prevalence of antepartum depression was also lower than the previously reported rates of 7–15%
Aralis, Shoptaw, Brookmeyer, Ragsdale, Bolan and Gorbach	2018	Los Angeles, CA	To identify specific social, structural, and psychiatric factors that are associated with attainment of viral suppression among a sample of urban-dwelling HIV-positive MSM	155	Patient Interviews	Findings provide support for comprehensive intervention programs that emphasize prevention and treatment of cigarette, methamphetamine, and other drug use, and promote improved connection to psychiatric care
Beer, Tie, Padilla, Shouse, and Medical Monitoring Project	2019	United States	To estimate the prevalence of GAD symptoms among adults diagnosed with HIV in the United States to inform effective HIV prevention care and effort	3654	The Medical Monitoring Project survey	GAD symptom prevalence among PLWH was higher than among the general US population, indicating an excess burden of anxiety among PLWH. Outcomes along the HIV care continuum were poorer for people with anxiety, and the risk for HIV transmission was higher among persons with anxiety. Incorporating routine screening for GAD in HIV clinical settings may help improve health outcomes, reduce HIV transmission, and save healthcare costs
Belenky, Pence, Cole, Dusetzina, Edmonds, Oberlander, Plankey, Adedimeji, Wilson, Cohen, Cohen, Milam, and Adimora	2019	Six study sites located in Bronx, NY; Brooklyn, NY; Washington, DC; San Francisco, CA; Los Angeles, CA; Chicago, IL	To estimate the effects of Medicare Part D implementation on antidepressant use, depressive symptoms, and hospitalization among women with HIV	801	A secondary data analysis of claims data	The study provides no evidence of dual eligible HIV patients’ decreased use of antidepressant after the implementation of Medicare Part D. Dual eligible people may have had stable medication use, better access to medical care through Medicare both before and after Medicare Part D
Bengtson, Pence, Mimiaga, Gaynes, Moore, Christopoulos, O'Cleirigh, Grelotti, Napravnik, Crane, and Mugavero	2019	United States	To find associations between viral load, depression, and appointment adherence for HIV care	1057	Prospective cohort study	Impaired mental health adversely affected engagement in care along the HIV treatment cascade. Recent depressive symptoms are a risk factor for unsuppressed viral load. Preexisting mental health conditions may influence HIV appointment adherence
Brown, Qian, Harrison, Haider, Conserve, Deming, Zhang, and Li	2021	South Carolina	To assess if depressive symptoms mediated the association between sexual assault and ART adherence among adults	326	Cross Sectional Survey w/ Mediation Analysis	Interventions addressing depressive symptoms may improve ART adherence among adults aged 18–34 and 35–49 and men. Programs also addressing depressive symptoms and using trauma-informed approaches may improve ART adherence, especially among middle-age populations, men, and women
Carney, Daniels, Xu, Sunil, Ganesan, Blaylock, Kronmann, Schofield, Lalani, Agan, and Okulicz	2021	United States	To identify associations between depression and HIV treatment outcomes in a male US military population with HIV infection	549	Prospective cohort study	There were no observed associations between depression and self-reported medication adherence. However, participants with recent onset depression had lower odds of reaching viral load suppression
Cederbaum, Rice, Craddock, Pimentel, and Beaver	2017	Los Angeles County, CA	To explore the structure and composition of HIV-positive African American and Latina women’s social networks. To determine whether network processes were associated with mental health, disclosure, and support	46	Survey	Respondents reported that a significant proportion of network members (66%) were aware of participants’ HIV status, a higher rate of disclosure than expected given known challenges related to stigma and discrimination experienced by women due to their positive serostatus. Even with relatively high rates of disclosure, support from network members was limited. Participants with more network who made them feel loved also reported less fear related to disclosure
Choi, DiNitto, Marti, and Choi	2016	USA	To examine the associations of mental health and substance-use disorders with emergency department (ED) outcomes among PLWH in four age groups: 21–34, 35–49, 50–64, and 65+ years	115,656	Statistical analysis	The prevalence of mental health and substance-use disorders among PLWH are high and play a significant role in ED visit outcomes. Integrated care for PLWH and co-occurring mental health and substance-use disorders is necessary mental health and substance-use disorders to reduce ED visits and costly hospital admissions, especially for older adults with HIV
Cleland, Gwadz, Collins, Wilton, Sherpa, Dorsen, Leonard, Cluesman, Martinez, Ritchie, and Ayvazyan	2021	NYC, NY	To advance substance abuse literature and its relationship to critical risk factors among the population of African American/Black and Latino PLWH with low-SES backgrounds and who are engaged in HIV care below recommended levels and who also have low ART adherence	512	Cross-sectional and qualitative study	These results that show a link between mental health, comorbidities, and HIV care should be used among HIV policymakers, HIV care providers, and interventionists to improve quality of care. Improvements in engagement and treatment for African American/Black and Latino PLWH with barriers to engagement, polysubstance use, and co-occurring risk factors need to happen in clinical settings. Increased outreach and engagement can bring more AABL PLWH into care settings, and offer specialized services for screening, treatment, and retention
Coleman	2017	Philadelphia, PA	To determine which domains of health-related quality of life after controlling for demographic correlates predict depressive symptoms among seropositive African Americans on ART	70	Descriptive correlational study	Changes in HRQOL were related to depressive symptoms. Assessing for depression when there are functional changes in health-related quality of life is important. Future studies need to investigate how changes in health-related quality of life affect African Americans with HIV/AIDS from a longitudinal perspective. Longitudinal data could provide an understanding about the clinical trajectory that changes in health-related quality of life have on depressive symptoms
Coviello, Lovato, Apostol, Eisenberg, Metzger, Szucs-Reed, Kiryankova-Dalseth, Kelly, Jackson, Plano, and Blank	2018	Philadelphia, PA	To assess HIV load suppression among 254 psychiatric inpatients with comorbid substance use disorders in Philadelphia to determine if the population has greater difficulty achieving viral suppression	254	Secondary data analysis	The 52% viral load suppression rate among psychiatric inpatient was higher than expected, given that the CDC’s national suppression rate among those diagnosed with HIV in the general population is 58%
Dale, Safren	2018	USA	To improve medication adherence for Black women living with HIV	5	Case study	The case studies of five Black women living with HIV and histories of trauma who participated in treatment provide support for the use of the treatment
Dalseth, Reed, Eisenberg, and Blank	2018	Philadelphia, PA	To evaluate the interaction between psychiatric diagnosis and response to a community-based intervention targeted at treatment adherence in 236 HIV+ persons with co-occurring mental illness	236	Prospective cohort study	Findings suggest that successful adherence interventions should be informed by psychiatric symptomatology
Ge, McCaul, Nolan, Wei, Liu, and Chander	2019	Baltimore, MD	To determine the associations between alcohol use and anxiety and retention in care among women with HIV	364	Retrospective study	The study found that among women with HIV, frequency of alcohol use is independently associated with worse retention in care. This finding suggests an alcohol-related impairment of life behaviors and function among women with HIV who were not able to keep scheduled primary care visits
Gokhale, Weiser, Sullivan, Luo, Shu, and Bradley	2019	Atlanta, GA	To find associations between depression prevalence, anti-depressant treatment status and sustained viral load suppression among adults living with HIV in care in the US	26,150	Cross sectional survey w/ medical chart abstraction	The burden of depression among adults living with HIV in care is high, and while this study only minimally showed that depression was associated with a lower prevalence of sustained viral load suppression, diagnosing and treating depression in PLWH is crucial in order to improve mental health and avoid poor health outcomes
Goulding, Wilson, MaWhinney, Jankowski, and Erlandson	2020	Colorado	To examine the effects of exercise on depressive symptoms and quality of life among older people living with HIV	69	Randomized clinical trial	Older PLWH initiating an exercise intervention report lower QoL and more depressive symptoms when compared to uninfected peers. Mental health outcomes of PLWHs did not show significant improvements over the 24 weeks. Exercise alone may not be enough to result in consistent improvements in depressive symptoms or QoL in an older PLWH with a high burden of mental illness
Hutton, Cardin, Ereme, Chander, Xu, and McCaul	2020	Baltimore, MD	To compare sample prevalence of Axis-1 psychiatric diagnoses (Major Depressive Disorder, PTSD, and Bipolar Affective Disorders) using the Structured Clinical Interview for DSM-IV with those from general population African American women in the National Comorbidity Survey-Replication	315	Cohort study	African American WLWH enrolled in HIV clinical care, had a high prevalence of current and lifetime psychiatric disorders, more than twice the prevalence found among African American women in the general population
Jain, Li, Adams-Huet, Tiruneh, Luque, Duarte, Trombello and Nijhawan	2021	Texas, USA	To understand who was at risk for depression PLWH and if this risk varied between those who achieved HIV viral load suppression or not	5126	Retrospective, cross-sectional study	The study found that almost one out of four patients experienced moderate to severe symptoms of depression. When examining HIV-related factors, it was found that those who listed IDU or MSM as an HIV risk factor had a higher odds for depression as well as those with AIDS. Achieving HIV viral suppression and being engaged in care was found to reduce the odds of having depressive symptoms
Jang, Satre, Leyden, Leibowitz, and Silverberg	2019	North Carolina	To evaluate the independent effect of age on QoL among PLWH and examine the association between substance use, mental health symptoms, demographics, and HIV clinical characteristics and QoL in PLWH	614	Cross-sectional study	QoL in PLWH varies by age. Substance use, depression, and anxiety are associated with mental QoL. Injection drug use and depression are associated with physical QoL
Leddy, Zakaras, Shieh, Conroy, Ofotokun, Tien, and Weiser	2021	San Francisco and Atlanta	To examine the circumstances in which food insecurity and intimate partner violence co-occur and the mechanism through which their convergence influences HIV risk and treatment behaviors among women living with and at risk for HIV in the U.S.	24	Semi-structured interviews	Finding revealed that converging experiences of food insecurity and violence led to or exacerbate poor mental health conditions, and the majority noted turning to substances to help them cope. Poor mental health outcomes and substance use then continued to perpetuate the cycle of food insecurity, and violence. Ultimately, the co-occurring and mutually reinforcing interactions between food insecurity, intimate partner violence, poor mental health and substance use contributed to worse health outcomes by increasing HIV risk behaviors and undermining engagement in HIV care and treatment
Majeed, van Wijngaarden, Dolan, and Shah	2017	Rochester, NY	To examine the relationship between basic psychological needs, depression and quality of life in people living with HIV (PLWH)	65	Cross sectional study	Basic psychological needs and depression may be specific targets for psychosocial interventions aimed at improving quality of life in PLWH to promote successful aging
Mannes, Hearn, Zhou, Janelle, Cook, and Ennis	2019	Florida, USA	To examine the association between GAD symptoms and healthcare utilization (HCU) among 801 people living with HIV (PLWH)	801	Cohort study	Depression was associated with fewer ED/urgent care visits and overnight hospitalizations, while no association was found with missed primary care appointments. The role of anxiety in illness management remains understudied among PLWH. Anxiety identification and the development of interventions for anxiety among PLWH may have important consequences for healthcare cost saving, patient retention in care, and HIV-disease management
Milanini, Catella, Perkovich, Esmaeili-Firidouni, Wendelken, Paul, Greene, Ketelle, and Valcour	2017	San Francisco, CA	To compare the burden of neuropsychiatric symptoms of older PLWH with cognitive impairment and without cognitive impairment	74	Cross-sectional study	Cognitive impairment among older PLWH were not associated with neuropsychiatric symptoms
Millar, Starks, Gurung, and Parsons	2017	New York City	To explore associations between substance use, depression, physical comorbid health conditions and quality of life among older PLWH	114	Cross-sectional study	Both mental and physical comorbidities are negatively associated with quality of life among older PLWH
Mitchell, Nguyen, Maragh-Bass, Isenberg, Beach and Knowlton	2017	Baltimore, Maryland	The objective of this study was to examine associations between chronic pain, depression, and current substance use, with patient-provider engagement among a sample of primarily urban African American PLHIV with a drug use history	377	Patient questionnaire	Patient-provider engagement was associated with better ART adherence, which was associated with higher viral suppression. Findings suggest the need for attention to patient-provider engagement in PLHIV
Momenzadeh, Shumay, Dong, et al	2021	San Francisco, CA	Administrative and claims data on inpatient psychiatric services to Zuckerberg San Francisco General Hospital	248	A retrospective, observational study	In an acute inpatient psychiatry setting in an urban HIV/AIDS epicenter, where psychotic disorders and brief and involuntary admissions were the norm, guideline-recommended ART regiments were prescribed at almost 60% of discharges by the end of the study. Future studies should explore interventions to increase ART for high-risk subpopulations with SMI, including younger individuals or those with brief inpatient psychiatry hospitalizations
Momplaisir, Aaron, Bossert, Anderson, Tatahmentan, Okafor, Kemembin, Geller, Jemmott, and Brady	2018	Philadelphia, PA	To evaluate the association between depression during pregnancy and HIV care continuum outcomes of pregnant and postpartum WLWH enrolled in perinatal case management	281	Retrospective cohort analysis	Pregnant WLWH with possible or definite depression had similar care continuum outcomes to those without depression. These findings likely reflect the beneficial effects of intensive perinatal case management to offset the negative impact of depression in pregnancy and postpartum
Padilla, Frazier, Carree, Luke Shouse, and Fagan	2020	U.S., Washington, D.C. and Puerto Rico	To estimate the prevalence of depression, substance use, and HIV risk behaviors among adults experiencing recent homelessness	28,275	Cross-sectional survey	Approximately 8% of HIV-positive adults in HIV care experienced recent homelessness—higher than the national HIV prevention goal of 5%. Homelessness, depression and substance use challenge HIV care engagement, ART adherence, and viral suppression
Paolillo, Pasipanodya, Moore, Pence, Atkinson, Grelotti, Grant, Heaton, and Moore	2020	USA	To examine longitudinal associations between depressive symptoms and neurocognitive functioning among people living with HIV	448	Prospective cohort study	High cumulative burden of depression is associated with worsening neurocognition among PLVH, which may relate to poo HIV-related treatment outcomes
Perry, Remmert, Psaros, Pinkston, and Safren	2019	Southern New England	(1) To understand how mental health comorbidities among PLWH might interfere with learning cognitive-behavioral principles in psychotherapy. (2) To elicit PLWHs’ opinions for future interventions	30	Qualitative interviews	Multiple psychosocial and structural problems might negatively affect PLWH’s HIV health and well-being. More comprehensive and flexible treatments are needed
Robinson, Knowlton, Gielen and Gallo	2016	Maryland, USA	The purpose of this study was to examine the presence and correlates of a potential latent syndemic in a cohort of African American PLHIV	383	Patient survey	The study results suggest that, among disadvantaged African American PLHIV, active substance use, mental illness, and familial conflict non-negotiation may be contributing to poor HIV medical outcomes, particularly among African American females. The results also suggest that these factors lend themselves well to the application of Syndemic Theory. Our latent class analyses (LCA) reveal that the ‘Substance Use, Mental Illness, and Familial Conflict’ Syndemic approach yielded four distinct classes of behavioral risk patterns within the study population
Sahota, Cakouros, Russell, Hassler, Blank, and Barg	2020	Pennsylvania, USA	To identify causal pathways between HIV infection and severe mental illness	26	Cross-sectional interviews	Attention to the directionality of effects between mental illness and HIV has important implications for anticipatory guidance for infectious disease specialists, primary care providers and public health practitioners as well as policymakers
Sauceda, Lisha, Neilands, Christopoulos, Mathews, Levison, Dennis, and Johnson	2019	USA	To determine if greater depressive symptoms or substance use can explain the disparity in viral suppression rates between Latino and non-Latino White patients in HIV care	3129	Self-report data and cross-sectional study	The data did not show that Latinos reported greater cognitive-affective depression, or they were at greater risk for alcohol use when compared to non-Latino Whites. Latino patients were more likely to report current use of cocaine, opiates, or amphetamines. Problems of depression and alcohol use are found at the same rates in Latinos and non-Latino Whites. Illicit drug use may be higher for Latinos in this setting. Viral suppression outcomes among Latinos may be better explained by information outside of EMR settings like health literacy, competing needs, lack of adequate health insurance, and language barriers
Siyahhan Julnes, Auh, Krakora, Withers, Nora, Matthews, Steinbach, Snow, Smith, Nath, Morse, and Kapetanovic	2016	USA	To assess whether PTSD was associated with immune dysregulation. To explore the association between PTSD and selected markers of inflammation and immune activation in a cohort of HIV-infected, virally suppressed individuals. The objective of the natural history study is to determine the eligibility of HIV-infected individuals for participation in other Neuro-HIV studies at the NIH Clinical Center	114	Prospective cohort study	A high prevalence of PTSD was identified in this cohort of HIV-infected adults who were virally suppressed. These results suggest that PTSD may be associated with immune dysregulation even among antiretroviral therapy-adherent HIV-infected individuals
So-Armah, Gupta, Kundu, Stewart, Goulet, Butt, Sico, Marconi, Crystal, Rodriguez-Barradas, Budoff, Gibert, Chang, Bedimo, and Freiberg	2019	USA	To investigate if depression increases mortality risk and if this this association is stronger among people living with HIV	129,140	Prospective cohort study	Depressive symptoms were moderately associated with all-cause mortality among US veterans with HIV but not among their counterparts without HIV infection. Depression diagnoses were modestly associated with mortality among those without HIV but not among those with HIV infection
Tatum and Houston	2017	Chicago, IL	The purpose of this study was to examine the relationship between depressive symptoms, two types of motivation (intrinsic and extrinsic), and adherence, with self-efficacy as a mediator	121	Cross-sectional study	Findings suggest that interventions using motivational techniques to build adherence among patients with varying levels of depressive symptoms should address the role of treatment self-efficacy to improve their effectiveness
Thurston, Howell, Kamody, Maclin-Akinyemi, and Mandell	2018	Memphis Metropolitan Statistical Area (MSA)	To assess the relationship between Substance Abuse, Violence, and AIDS/HIV adversities and depressive symptoms among mothers living with HIV, as well as the moderating effect of resilience on this relationship	55	Longitudinal study	Results highlight how co-occurring adversities exacerbate depressive symptoms and underscore the role of resilience as a key protective factor among mothers living with HIV. Resilience could be a target of strengths-based treatment to reduce the negative effects of Substance Abuse, Violence, and AIDS/HIV on depressive symptoms among mothers
Todd, Cole, Pence, Lesko, Bacchetti, Cohen, Feaster, Gange, Griswold, Mack, Rubtsova, Wang, Weedon, Anastos, and Adimora	2017	USA	To estimate joint effects of antiretroviral therapy initiation and depressive symptoms on time to death	848	Prospective cohort study with semiannual follow-up	The study found a protective effect of antiretroviral therapy initiation on mortality, as well as a harmful effect of depressive symptoms, in a cohort of WLWH. The findings support the importance of effective treatment of depression among women with HIV. Among patients with or without depressive symptoms, antiretroviral therapy initiation and adherence is clearly critical. The study did not find evidence of interaction between these two common exposures on either the multiplicative or additive scale, in accordance with previous work
Tolson, Richey, Zhao, Korte, Brady, Haynes, and Meissner	2018	South Carolina, USA	To examine the associations between substance, use and mental health diagnoses, hospitalizations, and virologic suppression among patients at a large academic outpatient HIV clinic, Medical University of South Carolina (MUSC), in South Carolina	801	Retrospective medical record review	Study findings demonstrate that substance use, and mental health diagnoses are common among PLWH receiving care at MUSC. Moreover, substance use, and mental health diagnoses are associated with increased rates of hospitalization and lower rates of viral load suppression. Screening and treatment of substance use disorders will help improve retention and outcomes on the HIV care continuum
Tsuyuki, Pitpitan, Levi-Minzi, Urada, Kurtz, Stockman, and Surratt	2017	South Florida	To measure syndemic substance use disorder, violence, and mental health and compares the syndemic among HIV-infected heterosexual men, heterosexual women, and men who have sex with men (MSM)	481	Structured interviews	Study findings demonstrate that substance use disorder, violence, and mental health make-up a syndemic affecting PLWH. This syndemic was found to affect different sub-populations differently, suggesting that tailored intervention is necessary by health care providers
Willie, Overstreet, Sullivan, Sikkema, and Hansen	2016	NYC, NY	To examine whether distinct symptom components of each of these mental health problems uniquely influenced HIV medication adherence among WLWH who have experienced Childhood Sexual Abuse (CSA)	85	Secondary data analysis of a larger study of a randomized clinical trial	Analysis revealed that panic-related anxiety severity was significantly inversely related to HIV medication adherence. WLWH who have experienced CSA who report high levels of panic-related anxiety were less likely to be 90% adherent to HIV medications compared to women reporting low levels of panic-related anxiety
Yellin, Beckwith, Kurth, Liu, Castonguay, Patrick, Trezza, Bazerman, and Kuo	2018	Rhode Island, USA	The goal of this study was to assess the clinical outcomes of criminal justice involved populations on HIV-related syndemics of co-occurring and mutually reinforcing psychosocial conditions	106	Randomized controlled trial testing	Mental illness and substance use were concentrated in this sample, indicating a need for integrated care services
Young-Wolff, Sarovar, Sterling, Leibowitz, McCaw, Hare, Silverberg, and Satre	2019	San Francisco, CA	To examine the association between adverse childhood experiences, depression and anxiety symptoms, substance use, and HIV-related outcomes among PLWH at risk for unhealthy alcohol use and enrolled in a primary care-based alcohol intervention study	584	Cross-sectional study	PLWH disproportionately experience unhealthy drinking, substance use, anxiety and mood disorders, and have worse quality of life relative to the general population. Adverse childhood experiences—abuse (sexual, emotional, or physical), neglect, parental loss, exposure to parental mental illness, substance misuse, and domestic violence—are prevalent among PLWH and may constitute a risk factor for anxiety and poorer overall mental health. Trauma-informed interventions may reduce the negative health outcomes associated with adverse childhood experiences. Further research is needed to identify and test interventions to improve mental health outcomes among PLWH

## Results

The initial literature search resulted in a total of 1058 citations from the electronic databases. After removing 45 duplicates, a total of 1013 records were potentially eligible and screened. A full-text assessment of 61 articles was performed. This led to 45 eligible articles relevant to our systematic review that were included for final data extraction and further analysis. Figure 1 shows the flow chart of articles examined for this systematic review. The results begin with an overall summary of the studies included in the systematic review and then describe the findings related to different types of co-occurring mental health conditions that PLWH experience.

### Characteristics of Studies

**Fig. 2:**
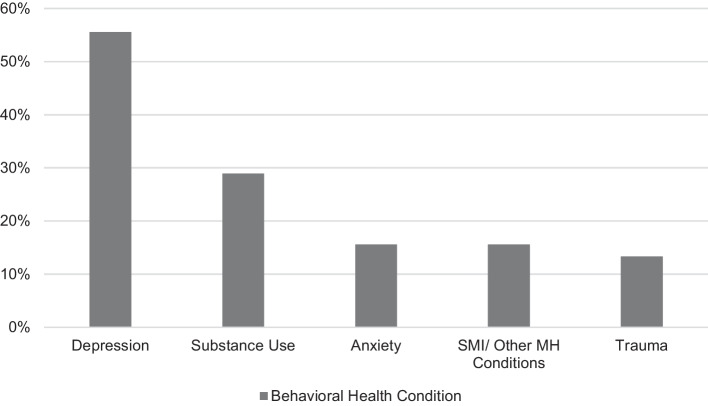
Studies by behavioral health diagnosis

Analysis of the 45 studies resulted in the identification of themes related to five major behavioral health categories (Fig. 2). Depression and substance use disorder were the most common conditions studied among people living with co-occurring HIV and mental health conditions, comprising 56% (25/45) and 29% (13/45) of studies reviewed, respectively. Other major behavioral health categories include anxiety at 16% (7/45), SMI and other psychiatric disorders at 16% (7/45), and trauma at 13% (6/45). Some studies conducted research across multiple behavioral health categories.

Cross-sectional (22/45, 46%) and cohort studies (14/45, 31%) were the major study designs. There were two randomized controlled trials (4%), one case study (2%), and seven (16%) secondary data analyses. For methodology, 96% (43/45) of the articles used quantitative methods. The sample sizes ranged from 5 to 129,140, with the smallest sample size belonging to a case study and the largest belonging to a national cohort study. The average sample size was 7178, and the median was 315. The cohort studies and cross-sectional studies mainly recruited participants from clinical settings and collected data through patient assessments. Studies with medium to large samples were usually recruited from or were embedded in existing large cohort studies. The two randomized controlled trials collected primary data from trials. The secondary data analyses used data from medical chart/medical record reviews, existing large cohort studies (Women’s Interagency HIV study, Preventing AIDS Through Health for Triples Study, etc.), HIV surveillance systems (e.g., the Medical Monitoring Project), or a large randomized clinical trial. The case study recruited participants from community clinics and community events.

The studies employed a variety of instruments to measure the dependent and independent variables. Demographic variables, which include social determinants of health (e.g., unemployment, housing, incarceration), and HIV-related behaviors (e.g., substance use, appointment adherence, care utilization) were mostly measured with self-report questions. Some studies measured substance use with standardized tests [19, 20]. Psychological and physical well-being variables were measured with verified scales (e.g., GAD, CES-D, PHQ-9, SF-12, NPI-Q, etc.). HIV-related outcomes (viral suppression, treatment adherence) were measured using biological markers or self-reports. Other outcome variables include patient/provider relationship, hospitalization, and emergency department/ urgent care visits, etc.

### Depression

Depression was the most consistent theme identified. Over half of the studies (55%) reviewed focused on depressive symptoms or a depression diagnosis among PLWH. Almost one in four PLWH experienced moderate to severe symptoms of depression [21, 22]. Although certain studies found no observed associations found between depression and self-reported adherence [21] or no direct association between depression and VLS [19, 23]. PLWH with recent onset depression were less likely to reach VLS and had increased mortality [21, 24]. This suggests that clinicians should be aware of changes in the mental health of their HIV patients and how this may impact HIV-related outcomes. Risk factors including female gender, Caucasian race, injection drug use, MSM, having ≥ 1 ED visit, poor appointment adherence, an AIDS diagnosis, or a positive drug screen by Substance Abuse and Mental Illness Symptoms Screener (SAMISS) increased PLWH’s odds for depression [22]. It is important to note the higher potential for depressive symptoms found among females and people who inject drugs and consider this as a topic for further investigation in future studies of health outcomes among PLWH and co-occurring mental health conditions.

Studies also suggest that changes in the severity of depressive symptoms may be more specifically related to social determinants of health than the patient-provider relationship for PLWH [23, 25–30]. Multiple social determinants of health such as lower income level, transactional sex, housing instability, long-term survivor status, low social support (perceived or real), poor educational attainment, and food insecurity were shown to be associated with a higher prevalence of depressive symptoms [23, 25–30]. Although the patient–provider relationship was beneficial for mental health outcomes in PLWH, addressing sociodemographic factors may be of greater importance [25]. Women who reported more individuals who could care for them had more family support and those who reported feeling loved were less likely to report stigma [28]. This highlights the importance of addressing social determinants of mental health such as social support and networks to better inform interventions to support the mental health of all PLWH, particularly WLWH. Additionally, PLWH with household incomes at or below the federal poverty level had a higher prevalence of depression compared with those living above the poverty level [29]. This can create additional barriers to diagnosing and treating depression in PLWH such as poor adherence to medical appointments due to lack of transportation or inability to pay insurance copayments, all of which ultimately impact the ability of a PLWH with co-occurring depression to achieve VLS. Moreover, the literature highlighted the situation where social determinants of health interact with common comorbidities of depression, including substance use, violence, and other traumatic experiences [20, 31, 32]. For example, Thurston et al. [20] suggested that experiencing more than one substance abuse, violence, and an HIV/AIDS diagnosis (SAVA) condition was associated with more depressive symptoms, particularly for WLWH. Going beyond the SAVA Syndemic, a qualitative study found that intersecting experiences of food insecurity and intimate partner violence co-occur with poor mental health and substance use, negatively influencing HIV prevention and treatment outcomes [32].

African American PLWH, especially females, were shown vulnerable to Substance Use, Mental Illness, and Familial Conflict non-negotiation related to poor HIV clinical outcomes [31]. Although not statistically significant, African American females with a higher prevalence of mental illness were less likely than African American males to achieve VLS (67.1 vs. 65.9%, respectively) and more likely to utilize acute care services in the prior 6 months (53.9 vs. 48.5%, respectively) [31]. Mental illness prevalence plays a crucial role in determining VLS. The impact could outweigh that of the presence of syndemic factors. For example, in a study examining the influence of substance use, mental illness, and familial conflict syndemic among PLWH who inject drugs, individuals with the highest prevalence of mental illness (Class 2) had 4.6 times the odds of having an unsuppressed viral load than individuals with the highest Substance Use, Mental Illness, and Familial Conflict syndemic burden (Class 4) (OR 1.48, 14.29; 95% CI) [31].

Certain healthcare utilization behaviors act as protective factors against depression. Pregnant WLWH with possible or definite depression were more likely to have perinatal care than WLWH with no depression (63.6 vs. 39.2, P < 0.05), therefore reducing the likelihood of perinatal transmission [23]. This is likely because WLWH with depression benefited from more supportive services and intensive case management during the prenatal period regardless of intentional help-seeking for depressive symptoms. Resilience also emerged as a protective factor by reducing depressive symptoms among mothers living with HIV [23]. Interventions focused on reducing the social determinants of mental health for PLWH with depression may focus on increasing resilience as a protective factor. Though this study focuses on pregnant WLWH, it offers an opportunity to consider how consistent engagement in healthcare-including supportive services and intensive case management-can provide support for PLWH with co-occurring depression.

### Substance Use

Substance use was identified in approximately 29% of the studies (13/45). Findings showed that PLWH disproportionately experience unhealthy alcohol use, drug use, anxiety, mood disorders, and have a worse overall QoL relative to the general population [3–5]. Specifically, a history of injection drug use or hazardous alcohol use was associated with poorer physical QoL (difference = − 3.6, 95% CI − 6.6, − 0.6; P < 0.05); overall substance use was linked to poorer mental QoL. The influence of substance use differs by age, which may be due to the better physical QoL but worse mental QoL in younger PLWH [4]. More work is needed to identify age-specific interventions on substance use to improve QoL for PLWH.

Aralis et al. [2] found significant associations between cigarette smoking (P = 0.030, OR 0.48, 95% CI 0.24, 0.93) and general drug use (P = 0.002, OR 0.29, 95% CI 0.13, 0.64) with and poor VLS among men of color who have sex with men. Additionally, African American WLWH engaging in heavy/hazardous drinking experienced lower CD4 count (P = 0.008), ART usage (P = 0.002), and VLS (P ≤ 0.001) compared to those with moderate/non-drinking levels [3].

Mental illness and substance use have been reported as co-occurring and mutually reinforcing among PLWH [20, 31, 32]. Co-occurring substance use and mood disorders led to poor engagement in cognitive-behavioral therapy/psychotherapy, reducing treatment effectiveness [8]. Active substance use, along with mental illness and familial conflict, all contributed to poor HIV-related health outcomes among disadvantaged African American PLWH [31]. Ultimately, the co-occurring and mutually reinforcing interactions between food insecurity, poor mental health, and substance use contributed to worse health outcomes by increasing HIV risk behaviors and undermining engagement in HIV care and treatment [32]. Comorbid substance uses and poor mental health outcomes were associated with lower rates of VLS and increased rates of hospitalization [33–35]. For example, in a study examining the VLS rate among psychiatric inpatients who use drugs, VLS among psychiatric inpatients was 52%, compared to the Center for Disease Control (CDC) national suppression rate of 58% [9].

The frequency of alcohol use may also be strongly correlated with poor retention in HIV care, particularly among WLWH [33–35]. Alcohol use was also found to be common among WLWH with polysubstance use such as marijuana, and cocaine [2, 3, 33, 35]. For example, African American WLWH who are hazardous/heavy drinkers were more likely to engage in polysubstance use (marijuana, cocaine, and heroin) compared to African American WLWH who are moderate/non-drinkers (OR 2.70, 4.39, 4.43, respectively; 95% CI) [3]. Engaging and treating African American, Black, and Latino PLWH who are polysubstance users along with comorbid risk factors is essential to ending the HIV epidemic.

Additionally, screening and treatment of substance use disorders can help improve retention and outcomes on the HIV care continuum [2, 3, 34–37]. Among a high-risk sample of PLWH, 52% achieved viral suppression, but recent opioid users were six times more likely to be virally unsuppressed than non-opioid users (OR 6.0; CI 1.1–31.7, P = 0.035) [9]. In a sample of MSM living with HIV, polysubstance users were 58% more likely to smoke cigarettes relative to non-polysubstance users [2]. Cigarette smoking (adjusted OR 0.48, 95% CI 0.24–0.93) and other drug use (adjusted OR 0.29, 95% CI 0.13–0.64) significantly decreased the likelihood of achieving viral suppression [2].

### Anxiety

Anxiety disorders were studied among PLWH in 16% of the studies reviewed. Findings showed that PLWH were disproportionately affected by anxiety [5, 38]. Moreover, PLWH with anxiety were more likely to experience co-occurring physical and behavioral health conditions, including mood disorders, unhealthy alcohol use, substance use, and poorer QoL [4, 5, 33, 39]. Cis-gender women were found to be disproportionately affected by the syndemic of co-occurring HIV, anxiety, and other conditions [33, 39].

The literature found that PLWH with anxiety experienced poorer health outcomes. Anxiety among PLWH was linked to lower sustained VLS in three studies [33, 38, 40]. Symptoms related to GAD were significantly associated with lower VLS (Prevalence Ratio (PR) = 0.87; 95% CI 0.80, 0.95) [38]. GAD symptoms were associated with engagement in risky behaviors such as condomless sex while virally unsuppressed (PR = 1.50; 95% CI 1.08, 2.09) [38]. Additionally, people with co-occurring HIV and moderate to severe anxiety were more likely to experience poorer mental QoL (difference = − 10.3, 95% CI − 13.0, − 7.5; P < 0.001) [4].

Anxiety among PLWH was associated with significantly lower adherence and engagement in HIV medical care [33, 38, 40, 41]. People living with co-occurring HIV and GAD had significantly lower ART adherence (PR = 0.83; 95% CI 0.74, 0.92) and engagement in HIV care (PR = 0.90; 95% CI 0.82, 0.99) and were over three times more likely to have an unmet need for mental health services (PR = 3.27; 95% CI 2.61, 4.11) [38]. Additionally, anxiety symptoms among PLWH had strong associations with increased emergency room visits and hospitalizations [38, 41]. For example, PLWH with moderate to severe GAD symptoms were more likely to have two (OR 3.31, CI 1.99–5.49, P < 0.001) or three or more (OR 2.06, CI 1.16–3.66, P = 0.013) overnight hospital stays and two (OR 2.61, CI 1.67–4.10, P < 0.001) or three or more (OR = 2.44, CI 1.48–4.02, P < 0.001) emergency department/urgent care facility visits [41].

Finally, WLWH and moderate to severe anxiety were found to experience significantly poorer adherence to HIV medication (panic-related anxiety [OR 0.71, 95% CI 0.54–0.93]) [40] and decreased odds of attending primary care visits (OR 0.69, P = 0.03) [33]. Overall, the literature proposed that incorporating routine screening for anxiety in HIV clinical settings and developing interventions for anxiety among PLWH may help reduce HIV transmission and improve health outcomes, patient retention in care, and HIV-disease management [33, 38, 40, 41].

### Serious Mental Illness and Other Mental Health Conditions

Themes related to SMI and other psychiatric conditions were studied among PLWH in 16% of the studies reviewed. Overall, study purpose and findings varied significantly, demonstrating a need for further research related to SMI among PLWH. HIV infection interacts with mental health symptoms among PLWH living with co-occurring SMI. A qualitative study on the causal pathways between HIV infection and SMI [42] found that HIV diagnosis often preceded depressive symptoms for people with unipolar depression (n = 11) and symptoms of mania and psychosis often preceded HIV for people with schizophrenia/schizoaffective and bipolar disorder (n = 15).

Further, SMI negatively affects HIV treatment outcomes. Five studies looked at HIV treatment and/or health outcomes among PLWH and co-occurring SMI, with four looking at behavioral health associations with HIV treatment adherence and/or VLS [2, 3, 9, 43], and one evaluating ART prescription patterns [44]. Two of these studies focused on PLWH who were receiving inpatient mental health treatment [9, 44]. An evaluation of ART prescription patterns for 506 PLWH receiving care within psychiatric inpatient settings indicated that ART was prescribed upon discharge for 39% of the study population, starting at 28% the first year and was generally increasing over time (χ2 6 = 14.05; P = 0.03) [44]. Findings from community-based treatment adherence interventions showed that PLWH with co-occurring psychotic and bipolar disorders had worse VLS compared to those with co-occurring non-psychotic depressive disorders [43].

### Post-traumatic Stress Disorder and Trauma

Themes related to trauma were studied among PLWH in 13% of the studies reviewed. Findings showed that PLWH had a higher prevalence of PTSD [3, 45] and were more likely to experience adverse/traumatic events compared to populations without HIV [5, 27]. For example, Hutton et al. reported that African American WLWH were three times more likely to experience lifetime PTSD, regardless of alcohol use ([heavy drinking OR/CI 6.27 (3.56, 11.0); P ≤ 0.001] [non-drinking OR/CI 4.48 (2.71, 7.40); P ≤ 0.001]) [3]. HIV self-care behaviors, such as taking ART medication, could be a trigger for traumatizing lived experiences such as victimization, internalized HIV stigma, and events that may have contributed to their current HIV status [40].

The impact of PTSD and trauma on health outcomes and treatment adherence varied by study. Brown et al. reported a significant association between sexual assault and depressive symptoms (P = 0.002) and ART non-adherence (P = 0.006) [27]. However, Young-Wolff et al. examined the association between adverse childhood experiences, depression and anxiety symptoms, substance use, and HIV-related outcomes among PLWH at risk for unhealthy alcohol use and found that adverse childhood experiences specifically were not associated with depression, substance use, or HIV-related outcomes [5]. Even when PLWH were adhered to ART, PTSD was still significantly associated with immune dysregulation (P = 0.03) [45].

Programs using trauma-informed interventions may improve ART adherence [27] and negative health outcomes associated with past or current experiences of trauma [5], especially among middle-aged populations, men, and women. Findings also support a tailored cognitive behavioral treatment approach in helping to improve HIV medication adherence and decrease PTSD symptoms [46] (41). Interventions should be tailored to address the stressors, challenges, and resiliencies of specific populations, specifically black women living with HIV [3, 46].

## Discussion

This review describes the demographic and clinical characteristics and patterns of engagement in the HIV and behavioral health care continuum among people living with co-occurring HIV and mental illness in the United States. This systematic review highlights an abundance of research focusing on depression and substance use in relation to health outcomes in PLWH. The extent to which the subcategories of anxiety, trauma, and serious mental illness have been studied is similar in terms of their overall proportion of the literature, but they make up a substantially lesser volume than both depression and substance use. Additionally, most of these studies focus on a single mental health condition. Findings from the study are consistent with previous research [47, 48], indicating a significant negative impact of mental health comorbidities on HIV outcomes.

### Overall Findings and Implications

Depression was the most prevalent comorbidity detected in the literature that interacts with HIV status. It both contributed to and was affected by HIV outcomes. Depression was associated with decreased HIV VLS [31]. Meanwhile, HIV risk behaviors and poor adherence predicted higher rates of depression among PLWH [22]. Our findings also suggested associations between multiple social determinants of health and depression in PLWH, which has implications for potential interventions targeting social determinants of health to improve depressive symptoms and HIV outcomes in PLWH. Similar results were found with anxiety, which is consistent with the general association between depression and anxiety [49]. However, anxiety and its specific role in HIV care and management remain understudied among PLWHA, indicating a further need for research in this area and work on the identification of anxiety as well as the development of interventions for anxiety among PLWHA.

Another highlight of the findings is that substance use often co-occurs with mental illness in a mutually reinforcing pattern in PLWH [20, 31, 32], which then contributes to decreased viral suppression, increased hospitalization, and decreased quality of life [33–35]. Particularly, excessive alcohol use was associated with poor retention in the case of WLWH [33–35]. Screening and treatment of polysubstance use disorders in PLWH and alcohol use interventions targeting WLWH can potentially help detect co-occurring mental illness in PLWH, to break the cycle of substance use and mental illness that leads to poor HIV outcomes.

Findings are less consistent regarding SMI and other psychiatric conditions. A qualitative study showed PLWH with non-psychotic depressive disorders had better VLS than those with psychotic and bipolar disorders [43]. Whereas a study on PLWH receiving inpatient psychiatric care found no association between psychiatric disorders and VLS [9]. Further research is warranted to determine whether SMI and other psychiatric conditions are correlated with HIV outcomes in PLWH and how they interact with other co-occurring factors in influencing HIV outcomes.

Experience of PTSD was affected by HIV status, while trauma predicted worse HIV outcomes through ART non-adherence or immune dysfunction [5, 45]. Existing trauma-informed interventions were shown to be effective in improving ART adherence and health outcomes associated with ACEs [5]. The dissemination and implementation of trauma-informed practices have potential benefits on HIV outcomes for PLWH. Veterans propose an interesting dilemma as they may show a high rate of PTSD and may require further consideration. Future studies should investigate the trauma-specific stressors, challenges, and resilience of specific PLWH populations who experienced trauma and PTSD [3, 46].

Moreover, the study found African American cisgender women disproportionately affected by the syndemics of HIV, substance use, anxiety, PTSD, and other mental health disorders. Thus, they had poorer HIV care adherence or VLS compared with other groups. Further studies should highlight the specific gender and racial factors in achieving optimal HIV outcomes among people living with co-occurring HIV and mental health conditions.

### Limitations

This research only includes articles from six chosen databases and has the potential to exclude research that may have been published with other databases. Most of the reviewed studies (46%) were cross-sectional studies, which could only suggest a correlation between mental health conditions and HIV outcomes with the direction of the effects undetermined. The literature mainly focused on the cisgender adult population and populations with higher risks, such as men who have sex with men. Other demographic groups such as the transgender, bisexual, non-binary, and teenager populations were under-represented, and this represents an area in need of further research. Although some literature defined the intersection of race and ethnicity with HIV/AIDS and mental health conditions as a focal point in their research, this was largely an understudied social determinant of health among the publications analyzed in this review. Additionally, the chosen articles measured the outcome variables with different instruments, which might render direct comparisons of the results problematic. This study also did not include grey literature which can be a useful resource in systematic reviews and the inclusion of grey literature in future reviews may add additional insight into this topic. Large-scale studies with similar outcome measurements will be needed for direct comparisons and more solid conclusions.

## Conclusion

This systematic review offers a comprehensive discussion on the co-occurring and mutually reinforcing behavioral health conditions of PLWH to provide meaningful insights into the syndemic effects of HIV, mental health, and substance use conditions. Specifically, this systematic review reconfirms that the majority focus of the literature on co-occurring HIV and mental and behavioral health conditions are focused on depression research, followed by substance use. There is a need for further research on anxiety, trauma, and other serious mental illness. Within the work on serious mental illness, there is a need to separate and define serious mental illness conditions (i/e bipolar disorder and schizophrenia). Future research should focus on a few key priority areas. Future studies should include strategies for investigating multiple mental health conditions rather than a single mental health condition in isolation. This strategy has already been employed to some extent by current studies included in this review but is generally not the major focus of the research. Next, there is a need to focus on the impact of social determinants of health on both mental health and health-related outcomes in PLWH. Finally, researchers should attempt to focus on studies that better understand and potentially identify causal mechanisms. This includes further investigations that can explore targeted interventions based on these demographic and clinical characteristics. Longitudinal cohort studies and clinical trials are needed to explore the causal relationships between mental health conditions and HIV outcomes.

Overall, effective HIV care should effectively evaluate and screen for mental health disorders and integrate interventions with strategies tailored to specific mental health conditions. Because efforts to end the HIV epidemic at the state and federal level emphasize the connection to care in PLWH [15, 16], it is essential to include effective mental health evaluation and care to improve the long-term quality of life among PLWH. More pointedly, the success in ending the HIV epidemic in the U.S. potentially depends on how effectively public health can break down the synergic effects of HIV infection, substance use, and mental health conditions. Based on this systematic review, future policies should allocate more resources towards integrated HIV and mental and behavioral health interventions that are tailored to meet the unique needs of PLWH.

## Data Availability

Not applicable.

## Abbreviations

ART: Antiretroviral therapy
GAD: Generalized anxiety disorder
HIV: Human immunodeficiency virus
MSM: Men who have sex with men
PLWH: People living with HIV
PTSD: Post-traumatic stress disorder
PRISMA: Preferred Reporting Items for Systematic Reviews and Meta-Analyses
QoL: Quality of life
SMI: Serious mental illness
VLS: Viral load suppression
WLWH: Women living with HIV
